# Steroids and Thyrotoxicosis Precipitate Periodic Paralysis

**DOI:** 10.7759/cureus.2106

**Published:** 2018-01-23

**Authors:** Rizwan Ahamed, Sarah McCalley, Anupam A Sule

**Affiliations:** 1 Internal Medicine, St. Joseph Mercy Oakland Hospital

**Keywords:** thyrotoxicosis, periodic paralysis, hypokalemia, corticosteroid, adverse drug reactions

## Abstract

Thyrotoxic Periodic Paralysis (TPP) belongs to a group of muscle diseases called channelopathies, which present with painless generalized muscle weakness without exertion. TPP can be precipitated by a large carbohydrate meal, stress, strenuous exercise, alcohol, a high-salt diet, menstruation, and cold temperatures. Rarely, steroids such as dexamethasone can also precipitate a TPP attack.

A 29-year-old Hispanic male, with a history of hyperthyroidism, presented to the emergency department with progressive weakness, predominantly in the lower extremities since morning. Earlier that day, the patient was seen in the same emergency department for difficulty in swallowing. He was diagnosed with uvulitis and received intramuscular dexamethasone and was discharged with amoxicillin for ten days. At home, he started to develop cramps in his lower extremities associated with paresthesias, which progressed to severe weakness to the point where he could not get out of bed. He returned to the hospital and revealed that he had suffered a similar episode following a steroid injection five years ago. He had not sought medical attention as it resolved spontaneously. He denied strenuous exercise, carbohydrate-rich meal, or alcohol ingestion. The patient had been noncompliant with atenolol and methimazole for the past month after losing his medical insurance. On examination, the patient appeared alert and calm. His vitals were significant for tachycardia of 123 beats per minute. Thyromegaly and tenderness were absent on examination of the neck. Muscle strength was 5/5 in the ankle dorsiflexors and ankle plantar flexors bilaterally, but the strength of the iliopsoas, quadriceps, and hamstrings was only 2/5 bilaterally. Deep tendon reflexes were diminished throughout to 1+. Laboratory findings were significant for profound hypokalemia, hypophosphatemia, low thyroid stimulating hormone, and elevated free T3 and T4 levels suggestive of hyperthyroidism. His electrolytes were replaced aggressively and his home medications were restarted. His electrolyte imbalance corrected and his symptoms resolved within a day and he was discharged home.

The overwhelming majority of TPP cases reported are male patients, hence this case demonstrates the need to be aware of this complication while treating hyperthyroid male patients with steroids. Hyperthyroidism potentiates catecholamine-mediated Na/K ATPase transport of potassium into the cells. Glucocorticoids are used in the treatment of thyroid storm as it prevents the peripheral conversion of T4 to T3. Moreover, glucocorticoids increase glucose levels stimulating insulin release, which shifts potassium intracellularly accentuating muscle weakness. Although the incidence of glucocorticoids causing TPP is low and not many cases are documented, it is still an important condition to be aware of and can have major clinical implications. Clinicians should be aware of this small subset of hyperthyroidism patients where the use of glucocorticoids can precipitate paralysis.

## Introduction

Periodic paralyses belong to a group of muscle diseases called channelopathies, which present with painless objective generalised muscle weakness without exertion [1]. Thyrotoxic periodic paralysis (TPP) is caused by an intracellular shift of potassium precipitated by increased levels of thyroid hormones. It is characterized by episodes of weakness and paralysis. TPP has been predominantly reported in young male patients of Asian descent [2]. It is extremely rare in other ethnicities [1]. A large carbohydrate meal, stress, strenuous exercise, alcohol, acute upper respiratory infection, a high-salt diet, menstruation, and cold temperatures have been reported to precipitate these attacks [1].

## Case presentation

A 29-year-old Hispanic male with a past medical history of hypertension and hyperthyroidism presented to the emergency department complaining of progressive weakness and paralysis. Earlier that same day, the patient had presented to the same emergency department and was treated for odynophagia due to uvulitis with amoxicillin 875 mg twice a day for ten days and a single intramuscular injection of dexamethasone 10 mg. Upon returning home, he started to develop cramps in his bilateral lower extremities that progressed to severe weakness to the point where he could not get out of bed. This was accompanied by paresthesias without sensory loss. He denied bowel or bladder incontinence. The patient had been unable to continue his atenolol 50 mg twice daily and methimazole 25 mg once daily for the past month after losing his job and medical insurance.

He denied any travel history and family history of similar episodes. Focused history taking revealed the patient had suffered from a similar episode following a steroid injection five years ago. He had not sought any medical attention at that time due to the transient and mild nature of the weakness.

On examination, the patient appeared alert, calm and in no acute distress. His temperature was 97.8 F, heart rate was 123 beats per minute, respiratory rate was 16 breaths per minute, oxygen saturation was 98% on room air, and blood pressure was 158/79 mmHg. Thyromegaly was absent, thyroid was non-tender and nodules were not palpable. Exophthalmos was absent. On neurological exam muscle strength was 5/5 in the ankle dorsiflexors and ankle plantar flexors bilaterally. Strength in the iliopsoas, quadriceps, and hamstrings was 2/5 bilaterally. Deep tendon reflexes of the quadriceps were 1+ bilaterally.

He was hypokalemic with a potassium of 2.0 mEq/L and hypophosphatemic with a phosphorus of 1.4 mg/dL. He did not have rhabdomyolysis with a normal creatinine kinase of 94 units/L. He was mildly acidotic with a bicarbonate level of 18 mEq/L. His random blood glucose was 242 mg/dL. He had hyperthyroidism with a significantly suppressed thyroid stimulating hormone (TSH) of <=0.03 miu/ml in the presence of elevated free thyroxine-4 (T4) of 2.97 nanogram/dL. Electrocardiogram showed normal sinus rhythm. He was given a total of 140 mEq of potassium (both oral and intravenously). Phosphate was replaced with 15 mmol intravenously.

The patient’s weakness improved and gradually resolved within 24 hours. The diagnosis of hyperthyroidism was confirmed with a low TSH and elevated T4. He was given two months of prescriptions for his methimazole and atenolol at the time of discharge. Based on the clinical presentation of acute onset hypokalemic muscle weakness in a patient with hyperthyroidism after the onset of corticosteroids and a history of a previous similar episode, a diagnosis of TPP episode induced by the injection of dexamethasone was established.

## Discussion

Thyroid hormone-mediated sensitization of adrenergic receptors leads to stimulation of the Na+/K+ ATPase channels in the skeletal muscles with a resultant influx of potassium into the cells. Thyroid hormone also regulates expression of the gene encoding Kir 2.6, the inwardly rectifying potassium channel which plays a part in the maintenance of resting membrane potential [3]. A mutation in this gene alters muscle membrane excitability, decreasing the ability of muscles to respond to stimulation and has been found in over a third of the patients with TPP.

A TPP episode can be precipitated by any stimulus which leads to the movement of potassium from the extracellular space into the intracellular space. Previously reported triggering factors include a large carbohydrate meal, stress, strenuous exercise, acute upper respiratory infection, a high-salt diet, alcohol, and cold temperatures [1]. Corticosteroids increase the number of Na+/K+ ATPase molecules in the skeletal muscle. Corticosteroids mediated hyperglycemia can lead to insulin secretion causing an intracellular shift of potassium. The resulting hypokalemia in a patient with thyrotoxicosis can precipitate a TPP attack if the patient has a mutation predisposing to periodic paralysis (e.g., Kir channelopathy).

Other causes of acute quadriparesis that should be considered are acute inflammatory demyelinating polyneuropathy, acute myelopathies (ascending pattern with predominant lower extremity features), botulism (predominant bulbar features), tick paralysis (travel history of return from the endemic area), and myesthenic crisis (usually known history). The hypokalemia serves as the initial indicator for TPP with hyperthyroid hormone profile providing additional supplementation of the diagnosis [1]. A very small proportion of patients actually have synchronous clinical features of thyrotoxicosis [2].

Out of 135 patients with TPP, only one was precipitated by steroids [2]. There have been three case reports of prednisone [4-6], two of methylprednisolone [7-8], and one of hydrocortisone [9] precipitating TPP. This is the first case report of an attack precipitated by a single dose of dexamethasone. Only one of the above patients presented with thyroid storm [7]. All of these cases had a coprecipitating factor. In the above case, the pharyngitis (respiratory tract infection) could be considered a co-precipitating factor.

## Conclusions

Glucocorticoids are commonly used for a variety of treatments. Physicians should be aware that the use of glucocorticoids in the presence of hyperthyroidism can precipitate TPP in a subset of patients.
